# Supplementary material for: Novel Ex Vivo DOAC Removal Methods Reduce Interference in Lupus Anticoagulant Testing
Source: Diagnostics (Basel). 2022 Oct 17;12(10):2520. doi: 10.3390/diagnostics12102520 (PMC9601296; doi:10.3390/diagnostics12102520)
Supplement: Supplementary file 1 [file diagnostics-12-02520-s001.zip › diagnostics-1942215-supplementary.pdf]

Supplementary material

Novel *ex vivo* DOAC removal methods reduce interference in lupus anticoagulant testing

Paula Savola, Marja Lemponen, Lotta Joutsi-Korhonen, Tuukka A. Helin

Supplementary Table S1. Coagulation tests and analyzers used in the study.

Test	Reagent(s)	Instrument
protein C activity (PC)	Berichrom Protein C, (OUVV15), Siemens Healthcare Diagnostics	BCS XP analyzer (Siemens Healthineers)
protein S free antigen	INNOVANCE Free PS Ag (OPGL03), Siemens	BCS XP analyzer (Siemens Healthineers)
activated protein C activity (APC)	Coatest APC Resistance kit (H-823120), Chromogenix (Diapharma)	BCS XP analyzer (Siemens Healthineers)
lupus anticoagulant APTT	Dade Actin FSL Activated PTT Reagent, Siemens REF: B4219-1<br>Dade Actin FS Reagent, Siemens REF: B4218-20	BCS XP analyzer (Siemens Healthineers)
lupus anticoagulant RVVT	LA 1 Screening Reagent, Siemens REF: OQGP17<br>LA 2 Confirmation Reagent, Siemens REF: OQGR13	BCS XP analyzer (Siemens Healthineers)
thrombin time	HemosIL Thrombin Time, (0020301700)	ACL TOP analyzers (Werfen)
prothrombin time	Owren´s PT (GHI31-20) (MediRox)<br>Owren´s Buffer (GHI150), (MediRox)	ACL TOP analyzers (Werfen)
APTT	HemosIL SynthASil Kit (0020006800)	ACL TOP analyzers (Werfen)
antithrombin activity	HemosIL Liquid Antithrombin Kit (0020030100)	ACL TOP analyzers (Werfen)
factor VIII activity	HemosIL Factor VIII Deficient plasma (0020011800), Werfen<br>HemosIL SynthASil (0020006800)<br>HemosIL Factor diluent, (0009757600), Werfen	ACL TOP analyzers (Werfen)
apixaban concentration	HemosIL Liquid Anti-Xa (0020302600), Werfen	ACL TOP 750 analyzer (Werfen)
rivaroxaban concentration	HemosIL Liquid Anti-Xa , (0020302600), Werfen	ACL TOP 750 analyzer (Werfen)
edoxaban concentration	HemosIL Liquid Anti-Xa (0020302600), Werfen	ACL TOP analyzers (Werfen)
dabigatran concentration	HemosIL Direct Thrombin inhibitor Assay (0020302800), Werfen<br>HemosIL Factor diluent, (0009757600), Werfen	ACL TOP 750 analyzer (Werfen)

Supplementary Table S2. Calibrators and control samples used in the study.

Name	Manufacturer	Ref.no	Sample matrix	Lot and expected concentration (ng/mL)
HemosIL Apixaban Low Control	Werfen, Bedford, USA	0020014300	lyophilized plasma	N0504105: 71<br>N0696475: 73
HemosIL Apixaban High Control	Werfen, Bedford, USA	0020014300	lyophilized plasma	N0504106: 292<br>N0696476: 300
HemosIL Apixaban Calibrator 2	Werfen, Bedford, USA	0020014200	lyophilized plasma	N1099450: 510<br>N0789031: 510
HemosIL Dabigatran Low Control	Werfen, Bedford, USA	0020013500	lyophilized plasma	N0797378: 49
HemosIL Dabigatran High Control	Werfen, Bedford, USA	0020013500	lyophilized plasma	N0797380: 187
HemosIL Dabigatran Calibrator 2	Werfen, Bedford, USA	0020013400	lyophilized plasma	N0889957: 454
Edoxaban Control 1	Diagnostica Stago, Asnières sur Seine Cedex, France	01072	lyophilized plasma	257742: 35-57<br>255059: 33-55
Edoxaban Control 2	Diagnostica Stago, Asnières sur Seine Cedex, France	01072	lyophilized plasma	257742: 89-129<br>255059: 87-127
Edoxaban Calibrator 2	Diagnostica Stago, Asnières sur Seine Cedex, France	01073	lyophilized plasma	256828: 29
Edoxaban Calibrator 3	Diagnostica Stago, Asnières sur Seine Cedex, France	01073	lyophilized plasma	256828: 92<br>254770: 87
Edoxaban Calibrator 4	Diagnostica Stago, Asnières sur Seine Cedex, France	01073	lyophilized plasma	256828: 138<br>254770: 134
HemosIL Rivaroxaban Low Control	Werfen, Bedford, USA	0020013700	lyophilized plasma	N0101543: 77
HemosIL Rivaroxaban High Control	Werfen, Bedford, USA	0020013700	lyophilized plasma	N0101544: 302
HemosIL Rivaroxaban Calibrator 2	Werfen, Bedford, USA	0020013600	lyophilized plasma	N0504114: 497<br>N0998445: 513

Supplementary Table S3. Coagulation test results in four patient samples without DOACs before and after DOAC removal using either DOAC Filter® or DOAC-Stop™.

	Before median (range)	After DOAC Filter® median (range)	After DOAC Stop™ median (range)
APTT, s	31 (30-48)	33 (30-53)	31 (29-48)
Prothrombin time, %	104 (17-142)	122 (20-161)	99.5 (16-133)
Thrombin time, s	23 (19-25)	22 (19-23)	24 (20-26)
APC, ratio	2.7 (2.3-2.9)	2.7 (2.2-2.8)	2.7 (2.4-2.9)
Antithrombin activity, %	107 (67-123)	133 (83-144)	103.5 (63-113)
Protein C activity, %	87 (48-149)	99.5 (53-135)	84.5 (47-147)
Protein S free antigen, %	91 (33-109)	95.5 (33-142)	88 (29-100)
Factor VIII activity, IU/dL	131 (111-229)	139 (109-220)	136.5 (109-227)

Abbreviations: APTT, activated partial thromboplastin time; s, seconds; APC, activated protein C.

Supplementary Figure S1. Lupus anticoagulant APTT test screening assay result correlations with DOAC concentration.

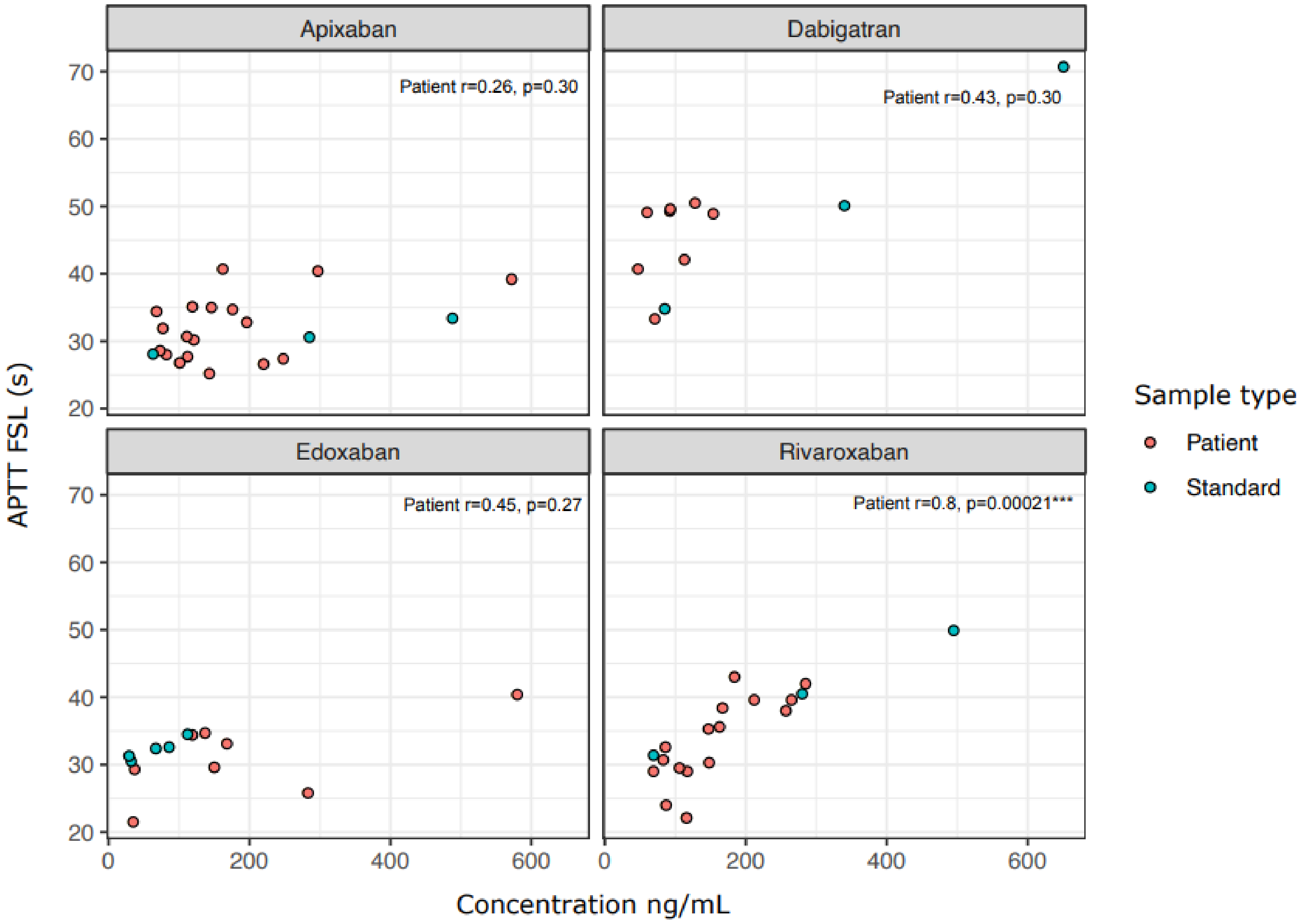

Results for the APTT-based FSL assay are shown prior treatment with DOAC-Stop™ or DOAC Filter®. Correlations were calculated using Spearman correlation and the p-value and Spearman rho are shown in the panels. Abbreviations: api, apixaban; dabi, dabigatran; edo, edoxaban; riva, rivaroxaban; conc, concentration as ng/mL.

Supplementary Figure S2. RVVT confirmation test clotting times reduce with DOAC Filter and DOAC Stop treatments.

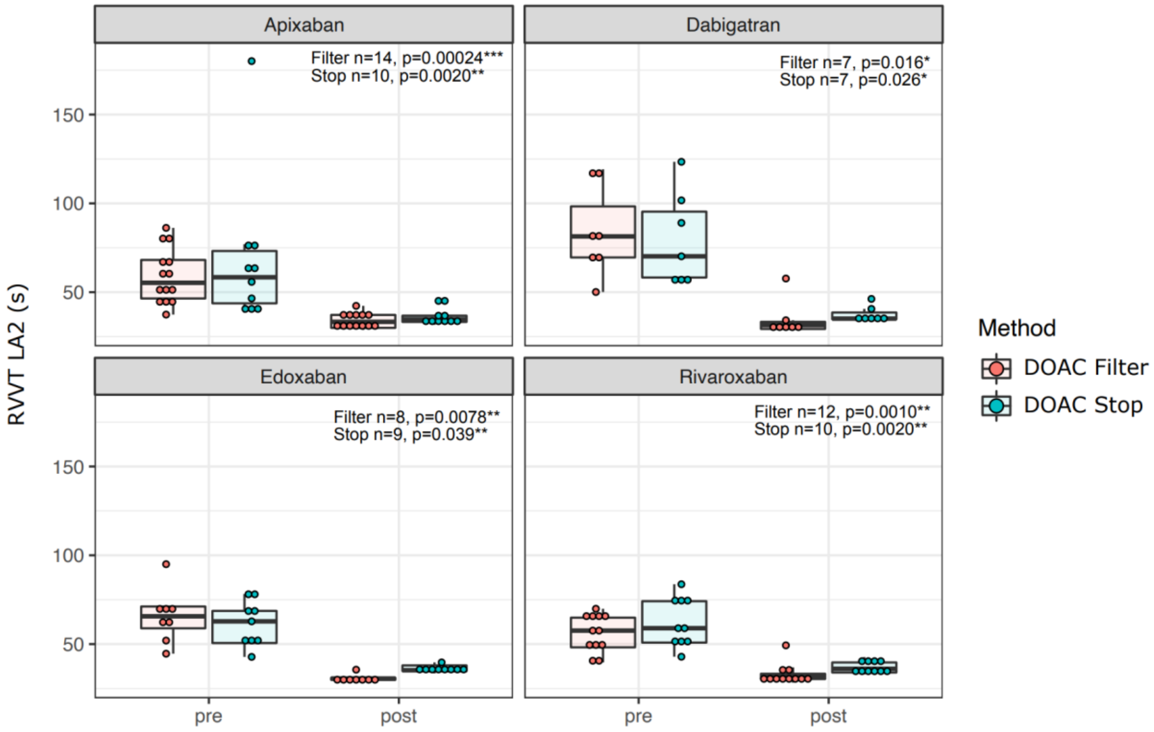

Statistical significance was tested with the paired sign test. Abbreviations: pre, before DOAC removal; post, after DOAC removal.

Supplementary Figure S3. APTT confirmation test clotting times before and after DOAC Stop and DOAC Filter treatments.

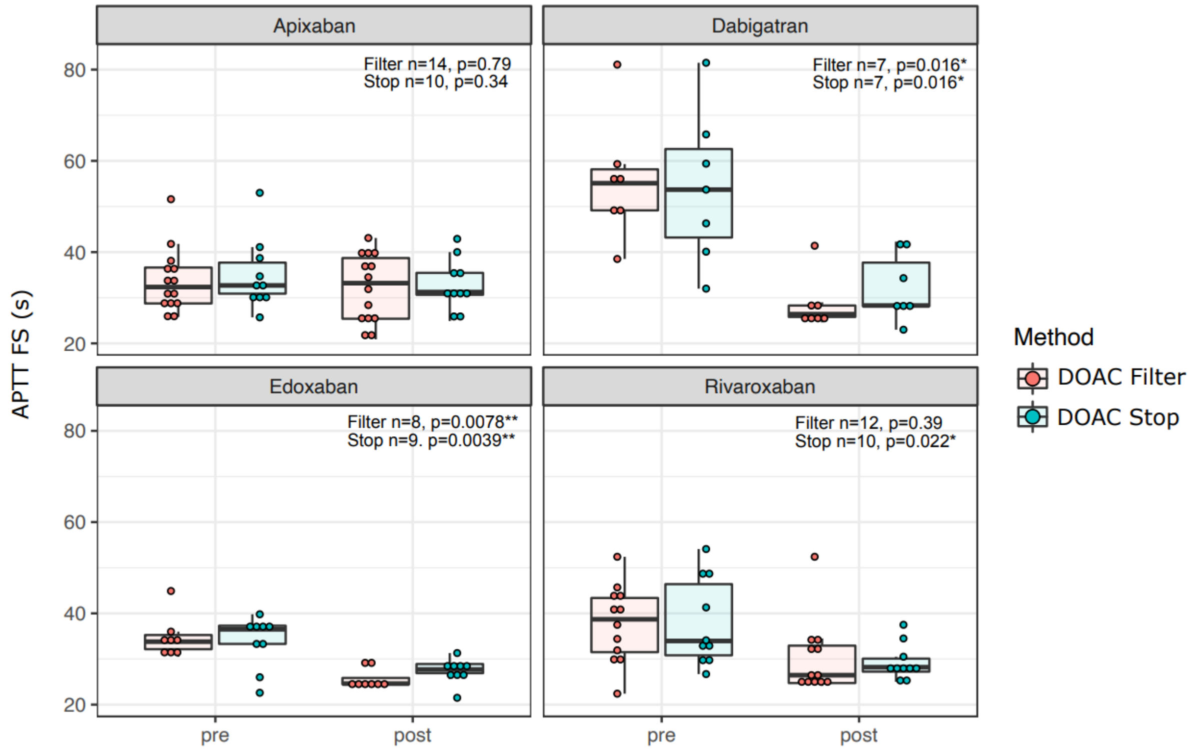

Statistical significance was tested with the paired sign test. Abbreviations: pre, before DOAC removal; post, after DOAC removal.

Supplementary Figure S4. Screening test results before and after DOAC removal.

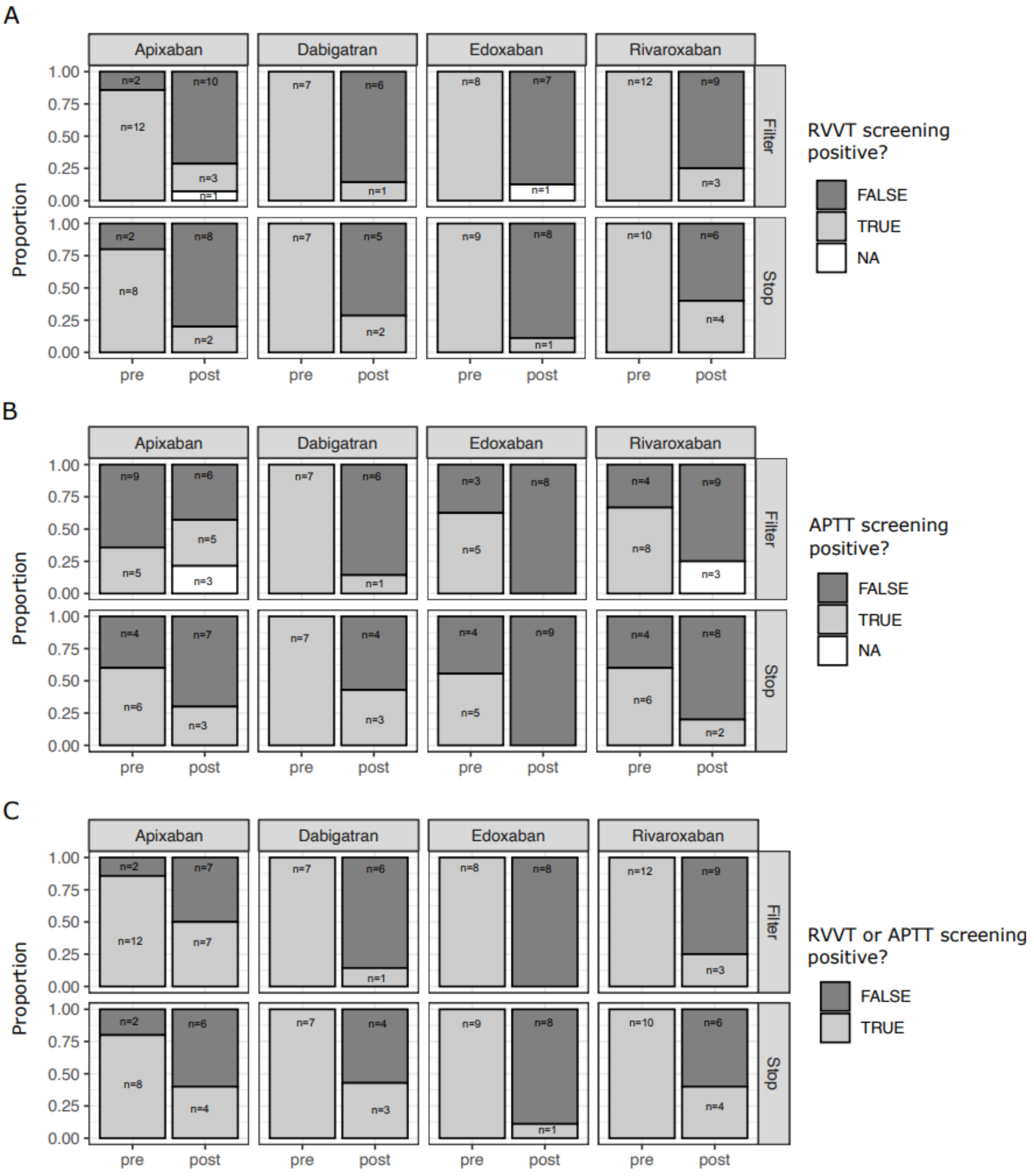

A. Results for the RVVT-based screening test assay. For statistical testing, all samples with DOAC were grouped but samples treated with DOAC Filter® or DOAC-Stop™ were separated in different groups for testing. p= 1.19e-07 for DOAC Filter® and p= 1.59e-06 for DOAC-Stop™ using the McNemar test. A positive screening test means that the sample would be subjected to mixing studies in a clinical laboratory setting, but the mixing studies were not performed in this study due to sample amount constraints. NA: not enough sample to determine a result. Same procedures were performed in panel B and C. B. Results for the APTT-based screening assay; p= 0.00080 for DOAC Filter® and p= 0.00041 for DOAC-Stop™. C. Results for grouped screening test results, i.e. if either screening test is positive, the sample will be categorized as having a positive

lupus. anticoagulant screening test result. p= 3.35e-07 for DOAC Filter® and p= 7.56e-06for DOAC-Stop™. In panel C, if the sample amount was sufficient to perform only one of the APTT or RVVT based screening tests, the sample was classified as positive if the single test performed was positive. If only one screening test was performed and it was negative, the sample was classified as negative in panel C.
